# SGLT-2 inhibitors and prevention of contrast-induced nephropathy in patients with diabetes undergoing coronary angiography and percutaneous coronary interventions: systematic review and meta-analysis

**DOI:** 10.3389/fendo.2023.1307715

**Published:** 2023-12-20

**Authors:** Edinson Dante Meregildo-Rodriguez, Martha Genara Asmat-Rubio, Gustavo Adolfo Vásquez-Tirado

**Affiliations:** ^1^ Escuela de Medicina, Universidad César Vallejo, Trujillo, Peru; ^2^ Escuela de Posgrado, Universidad Privada Antenor Orrego, Trujillo, Peru; ^3^ Escuela de Medicina, Universidad Privada Antenor Orrego, Trujillo, Peru

**Keywords:** sodium-glucose transporter 2 inhibitors, SGLT2 inhibitors, acute kidney injury, contrast-induced nephropathy, systematic review, meta-analysis

## Abstract

**Introduction:**

SGLT2 inhibitors (SGLT2Is) have demonstrated cardioprotective and nephroprotective effects in patients with and without diabetes. Recent studies suggest that SGLT2Is may reduce the risk of contrast-induced nephropathy (CIN) in patients with diabetes undergoing coronary arteriography (CAG) or percutaneous coronary interventions (PCI). However, the evidence is still inconclusive. We aimed to systematically review the evidence regarding the potential nephroprotective role of SGLT2Is in preventing CIN in this population.

**Methods:**

We searched for studies in six databases published up to September 30, 2023, following a PECO/PICO strategy. Initially, we meta-analyzed five studies, but due to several reasons, mainly methodological concerns, we excluded one RCT. In our final meta-analysis, we included four observational studies.

**Results:**

This meta-analysis comprised 2,572 patients with diabetes undergoing CAG or PCI, 512 patients treated with SGLT2Is, and 289 events of CIN. This is the first meta-analysis demonstrating that SGLT2Is may reduce the risk of developing CIN by up to 63% (RR 0.37; 95% CI 0.24–0.58) in patients with diabetes undergoing CAG or PCI, compared to not using SGLT2Is. Statistical heterogeneity was not significant (I^2^ = 0%, *p* = 0.91). We assessed the certainty of the evidence of this systematic review and meta-analysis, according to the GRADE criteria, as moderate.

**Conclusion:**

SGLT2Is significantly reduce the risk of CIN by up to 63% in patients with diabetes undergoing CAG or PCI. Clinical trials are needed; several are already underway, which could confirm our findings and investigate other unresolved issues, such as the optimal dose, type, and duration of SGLT2 inhibitor therapy to prevent CIN.

**Systematic Review:**

PROSPERO, identifier CRD42023412892.

## 1 Introduction

Diabetes mellitus and ischemic heart disease are growing public health problems worldwide (1–3). Contrast-induced nephropathy (CIN), also known as contrast-induced acute kidney injury (CI-AKI), is a common complication of coronary angiography (CAG) and percutaneous coronary intervention (PCI), especially in patients with diabetes. CIN is associated with high morbidity and mortality because it can lead to a significant decline in kidney function, and in severe cases, it can require dialysis. These complications also present with higher incidence in patients with type 2 diabetes mellitus (T2D) (4–6).

Sodium-glucose cotransporter-2 inhibitors (SGLT2Is) are a recent class of oral anti-diabetic agents (OADs) for treating patients with T2D (7). Currently, there exist four SGLT2Is approved by the Food and Drug Administration (FDA): empagliflozin, canagliflozin, dapagliflozin, and ertugliflozin (8). SGLT2Is act by inhibiting the renal reabsorption of glucose, enhancing renal glucose excretion, and decreasing serum glycemic levels (7). These medications reduce blood pressure, the risk of cardiovascular events (CVEs), and kidney disease in patients with and without diabetes (9–16). Two randomized controlled trials (RCTs), the DAPA-CKD (17) and the EMPA-Kidney (18) studies, showed that among patients with chronic kidney disease (CKD), regardless of the presence or absence of diabetes, gliflozins led to a lower risk of progression of CKD or death from renal o CVEs than placebo. Furthermore, the EMPEROR-Reduced trial showed that empagliflozin, compared to placebo, reduced deaths due to CVEs, heart failure (HF) hospitalizations, and the rate of decline in renal function in patients with and without T2D (19).

Likewise, the DAPA-HF trial found that patients with HF and a reduced ejection fraction treated with dapagliflozin had lower risks of worsening HF or death from CVEs than those who received a placebo, regardless of the presence or absence of diabetes; however, rates of worsening renal function was similar in both groups (20). In the DELIVER trial of patients with HF and mildly reduced or preserved ejection fraction, dapagliflozin reduced the risk of CV death or worsening HF regardless of baseline kidney function. Moreover, treatment with dapagliflozin slowed the rate of renal function decline, compared with placebo. Interestingly, this nephroprotective effect was more pronounced among patients with diabetes than those without (21).

Recent observational studies have suggested that SGLT2Is may prevent CIN in patients with diabetes undergoing coronary interventions such as CAG and PCI (22–26). However, observational studies showing discordant results also exist (27). A recent non-blinded open-labeled RCT failed to show a clear nephroprotective effect of SGLT2Is in patients undergoing PCI (28). Then, we aimed to conduct this systematic review and meta-analysis to evaluate the evidence on the effectiveness of SGLT2Is in preventing CIN in this patient population. The results of this study will provide important information on the potential role of SGLT2Is in the prevention of CIN in patients with diabetes undergoing coronary interventions.

## 2 Materials and methods

We conducted this systematic review according to the Cochrane Handbook for Systematic Reviews (29), PRISMA (30), and AMSTAR 2 (31) guidelines. We previously registered the protocol in PROSPERO (CRD42023412892).

We extensively searched MEDLINE (PubMed), Scopus, EMBASE, Web of Science, Google Scholar, and Cochrane Library. Each database was screened using thesaurus or controlled language terms (MeSH, Emtree, etc.), free terms, and their synonyms. These terms were combined using Boolean operators following our PECO/PICO strategy (*Population*: adult patients undergoing percutaneous coronary interventions or coronary artery bypass grafting; *Exposure*: treatment with any SGLT2I; *Comparator*: no use of SGLT2Is or other OADs; *Outcome*: Contrast-induced acute kidney injury OR Contrast-induced nephropathy).

The keywords included terms related to the exposure, such as “Sodium-Glucose Transporter 2 Inhibitors” OR “SLGT2 inhibitors” OR “Gliflozins”, and terms related to the outcome, such as “Contrast-induced nephropathy” OR “Contrast-induced acute kidney injury” OR “Contrast related acute kidney injury”. In addition, we conducted manual secondary screening of the references in primary and secondary studies. There were no restrictions on language or publication year. The search strategy is detailed in **Supplementary Materials**.

Our search included observational studies and randomized controlled trials (RCTs) published from inception until September 30, 2023. We excluded case reports, case series, and duplicated publications. All articles derived from the primary and secondary screenings were compiled using Zotero^®^ 6.0.15. After duplicate removal, these documents were imported into the Rayyan^®^ tool. Then, these records were screened and individually examined by two blinded and independent researchers (MGAR and GAVT). The studies were selected by consensus, and a third researcher acted as the arbitrator (EDMR) in case of discordance. All the articles collected were examined using the terms of the PECO/PICO strategy and the inclusion and exclusion criteria.

There is no consensus on the definition of CIN (32, 33). The criteria initially proposed by Barrett and Parfrey defined CIN as an absolute increase in serum creatinine levels by ≥0.5 mg/dL or a relative increase in serum creatinine by ≥25% from baseline within 72 hours after contrast exposure (34). In a recent meta-analysis, definitions of CIN based on serum creatinine levels ranged from 0.3 to 0.5 mg/dL for an absolute increase and 25 to 50% for a relative increase within 48-72 hours following intravenous contrast administration (35). Consequently, in this systematic review, we considered a patient to have CIN if they met any of the above definitions.

**Figure 1:**
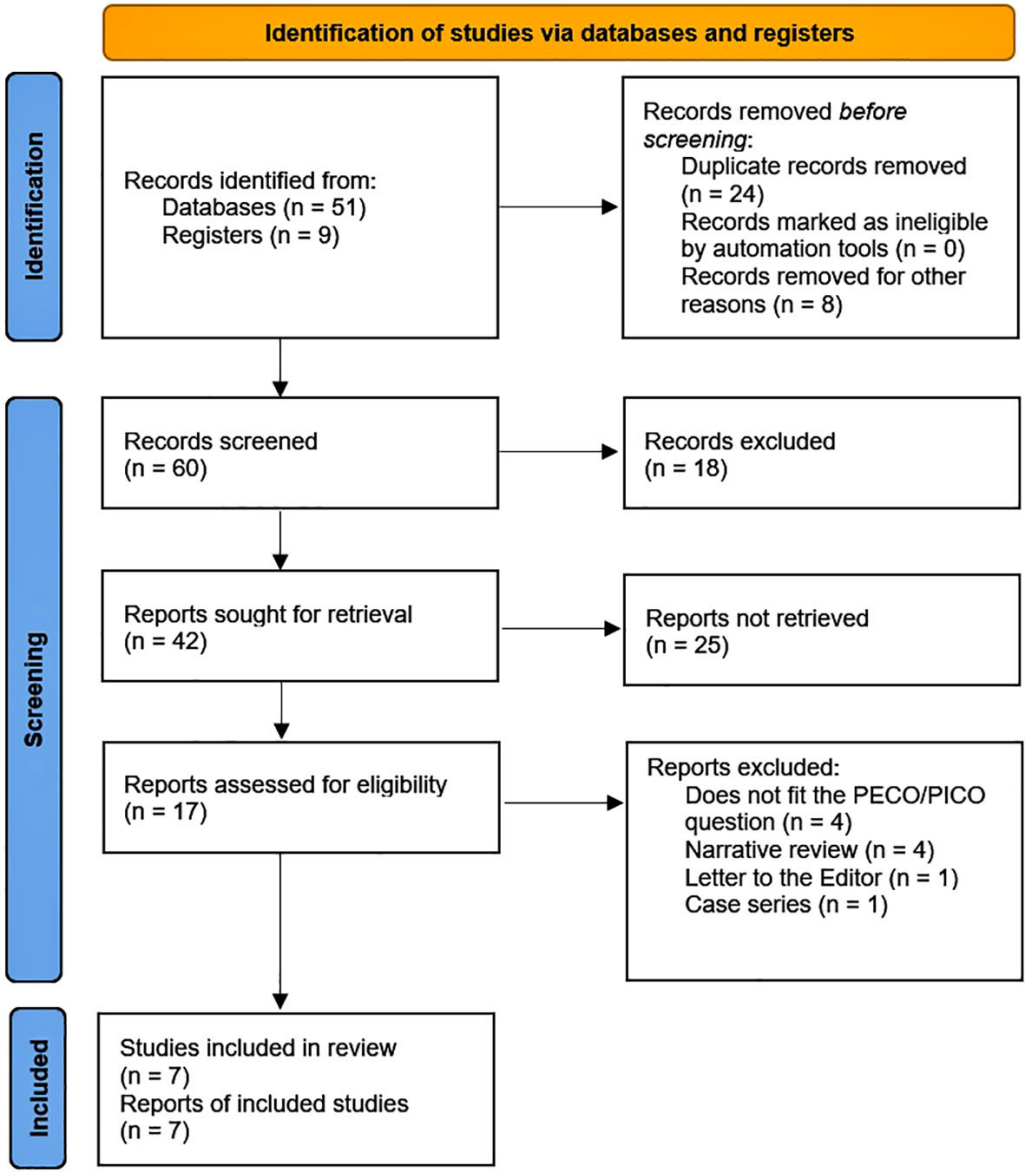
PRISMA 2020 flow diagram.

The selected papers were exported into a spreadsheet for a second full-text screening. The study selection process is detailed in **Figure 1**. For data extraction, the same two blinded and independent researchers performed the selection process, examined articles, and collected the relevant details of the study, such as the authors’ names, country and year of publication, the clinical and epidemiological characteristics of the population, the number of participants and cases, the measures of association, the confounding factors, and the outcomes. For the case of dichotomous and time-to-event variables, we compiled odds ratios (OR), relative risks (RR), and hazard ratios (HR) with 95% confidence intervals (95% CI). If critical data were missing, at least two emails were sent to the corresponding authors. Data from each paper were extracted and recorded in a spreadsheet. In case of a discrepancy, a third researcher (EDMR) solved it if necessary.

We pooled adjusted ORs, RRs, or HRs with 95% CIs using the generic inverse-variance method in the meta-analysis. We considered RRs equivalent to the ORs if the frequency of the event of interest was < 10% (36). We performed this meta-analysis using R^®^ 4.2.226 software. We summarized the quantitative synthesis using forest plots using the library *meta*, function *metagen*, and inverse variance method with *Restricted Maximum-Likelihood* (REML) for tau^2^. Our protocol stated that we would examine heterogeneity among studies with Cochran’s Q test and Higgins I^2^ statistic, using a fixed effects model if heterogeneity were not statistically significant (*p* > 0.10, I^2^ statistics < 40%). On the other hand, we would use a random effects model (29). The potential subgroups to be analyzed were study type, continent of origin, class, and dose of SGLT2I. We conducted sensitivity and influence analysis using the function *InfluenceAnalysis*.

We assessed the risk of bias using the Newcastle–Ottawa scale (NOS) (37) and version 2 of the Cochrane risk-of-bias tool for randomized trials (ROB 2) (38). We examined the publication bias using a funnel plot.

Two researchers (MGAR and GAVT) independently assessed the certainty of the evidence (CoE) of the study outcomes for each study outcome based on the Grading of Recommendations Assessment, Development, and Evaluation (GRADE) criteria (39, 40). Any reviewer discrepancy was resolved by discussion with the leading researcher (EDMR).

## 3 Results

We identified 60 records, 51 of which were retrieved from databases and 9 from registers. Among the latter, all were clinical trials, of which one was already published, and 7 were in different phases of execution, and 1 was suspended. After record removal and exclusion, we found 17 reports for retrieval and for assessing eligibility. Of these studies, 10 were excluded for different reasons (**Table S2**, **Supplementary Material**). Finally, we had 7 studies (22–28) included in our review (**Table 1**). Notably, two papers (22, 23) reported their outcomes with some differences, but the population included was the same. Consequently, in our meta-analysis of these two papers, we only considered the one that reported renal outcomes with more detail (23). Subsequently, we excluded the studies conducted by Bernardini F et al. (27) and Feitosa MPM et al. (28) for the reasons we explain in this section’s second and fourth paragraphs.

**Table 1:** General Characteristics of Included Studies.

Study, year, country	Design	Participants	Exposition	Outcome	Adjustment factors	OR/RR/HR (95% CI)
Paolisso P et al. (22) 2022, multicenter (Europe). The SGLT2I AMI PROTECT Registry.	PCS	AMI patients with diabetes undergoing PCI between 2018 and 2021. N = 646 AMI patients (with or without ST-segment elevation): 111 SGLT2Is users and 535 non-SGLT2Is users. Follow-up of 24 ± 13 months. The mean age of the overall population was 70 [61–79] years, and >77% were males. Exclusion criteria: patients on insulin therapy or with incomplete information on medical therapy, CABG, severe valvular HD, prosthetic heart valves, severe anemia, history of bleeding, pulmonary embolism, GFR <30 ml/min/1.73 m^2^, malignancies, and follow-up data shorter than 3 months. SGLT2I patients were younger (*p* < 0.001) and had better renal function at admission compared to non-SGLT2-I users (*p* = 0.886).	SGLT2Is users (started at least 3 months before hospitalization) vs. non-SGLT2Is users (if patients received other OAD strategies). The type and dose of SLGT2i are not specified. The sample size was powered to evaluate only a “class effect” but not the “doses effect.” The mean time of SGLT2I therapy duration was 7.3 ± 3 months. No explicit detail is provided if the SGLT2Is were suspended before the procedure and if this was the case, when they were restarted.	Primary endpoint: a composite of CV death, recurrent AMI, and hospitalization for HF (MACE). Secondary outcomes: i) in-hospital CV death, recurrent AMI, occurrence of arrhythmias, and CIN; ii) long-term CV mortality, recurrent AMI, and HF hospitalization. No definition of CIN is provided.	Not described for renal outcomes.	The use of SGLT2Is was an independent predictor of reduced risk of MACE (aHR = 0.57; 95% CI 0.33–0.99; *p* = 0.039) and HF hospitalization (aHR = 0.46; 95% CI 0.21–0.98; *p* = 0.041). CIN: among SGLT2Is users 6/111, 70/535 among non-SGLT2-I users (*p* = 0.022). Crude RR 0.4131; 95% CI 0.1841-0.9271.
Paolisso P et al. (23) 2023, multicenter (Europe). The SGLT2I AMI PROTECT Registry.				The development of CIN in patients with diabetes and AMI undergoing PCI. No definition of CIN is provided.	CKD and anti-diabetic therapy at admission (SGLT2Is vs. non-SGLT2Is users).	The use of SGLT2Is was an independent predictor of reduced rate of CIN (aOR 0.356; 95% CI 0.134–0.943, p = 0.038).
Hua R et al. (24) 2022, China	RCS	Patients with T2D undergoing PCI between January 1, 2020 and December 30, 2021. Before propensity score matching analysis: 245 in the SGLT2Is cohort and 1,265 in the non-user cohort. After matching: 242 SGLT2Is users and 242 non-users. Age: users 62.6 (55–63) and non-users 63.6 (57–71) years. Female: users 37 (30.5) and non-users 36 (29.8). Mean follow-up was not reported. Compared with non-users, SGLT2 inhibitor users tended to be at a lower age (*p* < 0.01), with significantly lower comorbidities, namely, HF (*p* < 0.01) and PCI history (*p* < 0.01), compared with SGLT2 inhibitor non-users. They also had higher eGFR (*p* = 0.0154) and a higher proportion of metformin use (*p* < 0.01) compared with non-users.	The exposure of interest was a prescription of an SGLT2I, including canagliflozin, empagliflozin, or dapagliflozin, for at least 6 months till the date of PCI. SGLT2Is types used: dapagliflozin 172 (71.1%), empagliflozin 41 (16.9%), canagliflozin 29 (12.0%). The dose of the SGLT2I is not specified. No explicit detail is provided if the SGLT2Is were stopped prior to the procedure and if this was the case, when they were restarted.	The risk of CIN in SGLT2I users undergoing PCI. The definition of CIN was based on the ESUR and KDIGO criteria. Evaluation of renal function indicators in users and non-users was performed at 24, 48, and 72 h post-PCI.	Age, sex, PCI history, NYHA ≥ III, and metformin use.	For CIN among users compared to non-users aOR 0.37; 95% CI 0.19–0.67; *p* < 0.01. CIN events: 13/245 in the SGLT2i user cohort and 121/1,265 in the non-user cohort (before propensity matching analysis). Age (aOR 1.06, 95% CI 1.02–1.11), PCI history (aOR 7.84, 95% CI 3.26–18.84), and NYHA grade ≥ III (aOR 7.92, 95% CI 1.80–34.91) were independent risk factors of CI-AKI for patients undergoing PCI.
Özkan U et (25). 2023, Turky	CCS	N = 312 patients with diabetes who underwent CAG and/or PCI between January 1 2020 and July 1 2022: 104 T2D patients using SGLT2Is and 208 T2D patients as a control group. The median age was 59.5 years (45–80) and 189 (60.6%) were male. Mean follow-up was not reported. Exclusion criteria: patients with active infection (including COVID-19), patients with CHF (EF < 40%), patients with malignancy, patients with autoimmune disease, patients using chronic anti-inflammatory drugs, patients with severe anemia, patients with eGFR < 30 mL/min/1.73 m^2^, patients who used nephrotoxic agents, patients with cardiogenic shock, and patients with a previous history of CIN. There were no differences between the treatment groups regarding sex (*p* = 0.54), age (*p* = 0.49) or preprocedural serum creatinine (*p* = 0.13) or eGFR (*p* = 0.28).	Patients who used SGLT2Is in the T2D treatment regimen before CAG and/or PCI were included in the study. The type and dose of SGLT2Is are not specified. The administration time of GLT2I prior to the procedure is not detailed. No explicit detail is provided if the SGLT2Is were stopped prior to the procedure and if this was the case, when they were restarted.	Diagnosis of CIN: a 0.5 mg/dL or 25% increase in SCr levels within 48 h, an increase in SCr of ≥ 1.5 times the baseline within 7 days, or a urinary output of < 0.5 mL/kg/h for at least 6 h after using the contrast agent compared to its level before the procedure (CAG and/or PCI). The values of SCr levels were measured before the procedure and 48–72 hours after that.	Age, sex, HT, smoking, prior revascularization, contrast volume, drinking, glucose, HbA1c, LVEF%, different medications, etc.	CIN: among no previous SGLT2Is users 64/208 and previous SGLT2Is users 14/104. SGLT2 inhibitors significantly reduced the risk of CIN aOR 0.41; 95% CI 0.142–0.966, *p* = 0.004.
Bernardini F et al. (27) 2022, Italy	RCS	N = 408 T2D patients undergoing PCI divided into three groups: 136 treated with new-antidiabetic drugs, 136 treated with standard-antidiabetic therapy, and 136 patients without diabetes. Mean follow-up was not reported. Exclusion criteria: not reported. The patients treated with SGLT2Is presented pre-PCI mean creatinine levels significantly lower (0.83 ± 0.16 mg/dL) than those treated with DPP-4 inhibitors (1.04 ± 0.34 mg/dL) and GLP-1 analogues (1.05 ± 0.39 mg/dL), (*p* for trend = 0.009).	New drugs: GLP-1 analogues, DPP-4 inhibitors, SGLT2Is. Standard therapy: metformin, sulfonylureas, thiazolidinediones, insulin. The type, dose, the administration time of SGLT2I prior to the procedure is not detailed. No explicit detail is provided if the SGLT2Is were suspended before the procedure and if this was the case, when they were restarted.	CIN was defined as an increase in SCr levels ≥ 0.3 mg/dL or > 25% of the base value, which occurred 24-48 hours after administration of the contrast medium. SCr level was measured at the time of hospitalization, 24-hour and 48-hour post-PCI.	None.	The incidence of CIN was 5.1% in the standard-antidiabetic therapy group, 3.8% in the new-antidiabetic drugs group, and 2.9% in patients without diabetes (*p* = 0.911). Crude RR 0.7143; 95% CI 0.2324-2.1953.
Santos-Gallego CG et al. (26) 2020, USA	RCS	Patients with diabetes who underwent PCI between January 2016 and May 2017. Patients with diabetes taking chronic SGLT2Is preadmission compared with age- and gender-matched patients with diabetes not taking SGLT2Is. Mean follow-up was not reported. Exclusion criteria: not reported. There was no difference in both groups in clinical characteristics (CVRF previous revascularization, HbA1c, LVEF, prior medical treatment), preprocedural analysis (serum creatinine, eGFR, BNP), or procedural characteristics (contrast volume, number of stents, LVEDP).	SGLT2Is users vs. non-SGLT2Is users matched by age and sex. The type, dose, the administration time of SGLT2I prior to the procedure is not detailed. No explicit detail is provided if the SGLT2Is were suspended prior to the procedure and if this was the case, when they were restarted.	CIN was defined as an increase in SCr higher than 30% or higher than 0.3 mg/dL one-day post-PCI	None.	Less SGLT2i users developed CIN (3.8 vs 17.3%, p<0.05) compared to non-users. Crude RR 0.2222; 95% CI 0.0504-0.9793.
Feitosa MPM et al. (28) 2023, Brasil. SAFE-PCI trial: a pilot, single-center, open-label RCT.	RCT	Patients with T2D undergoing elective PCI. Follow−up of 30 days. N = 42 patients (22 patients in the SGLT2Is group and 20 patients in the control group). The mean age among SGLT2Is users and control group were 65 ± 10 and 64 ± 6 years, respectively. Exclusion criteria: eGFR < 30 mL/min/1.73m^2^ or dialysis therapy, ACS in the last 30 days, need for urgent or emergency PCI, use of NSAID in the last 30 days before randomization; known pregnancy; and inability to sign the consent form. There were no differences between the treatment groups regarding sex (*p* = 0.42), age (*p* = 0.82), preprocedural serum creatinine (*p* = 0.24), urea (*p* = 0.64) or eGFR (*p* = 0.10).	SGLT2Is (empagliflozin 25 mg/d initiated at least 15 days before PCI and maintained until the end of the follow−up period) vs. placebo. SGLT2Is were continued throughout the follow-up period, including the day of the procedure.	CIN up to 48 h after PCI. Definition of CIN, according to KDIGO criteria.	Not described.	The incidence of CIN was similar in both groups: in 3 patients in the SGLT2Is group (13.6%) and 2 patients in the control group (10%) (*p* = 0.71). Crude RR 0.9680; 95% CI 0.7957-1.1776.

MC-CS, multi-center prospective cohort study; PCI, percutaneous coronary intervention; CABG, coronary artery bypass graft surgery; CAG, coronary arteriography; CHF, congestive heart failure; EF, ejection fraction; CV, cardiovascular; CVRF, cardiovascular risk factor; AMI, acute myocardial infarction; MACE, major CV events; AKI, acute kidney injury; CIN, contrast-induced nephropathy; CKD, chronic kidney disease; HF, heart failure; NSTEMI, non-ST segment Elevation Myocardial Infarction; CRP, C-reactive Protein; CyC, Cystatin C; HD, heart disease; Hs-TnI, high sensitivity Troponin I; MR, mitral regurgitation; GFR, glomerular filtration rate; eGFR, estimated GFR; ESUR, European Society of Urogenital Radiology; KDIGO, Kidney Disease, Improving Global Outcomes; PCS, prospective cohort study; RCS, retrospective cohort study; CCS, case-control study; RCT, randomized controlled trial; NYHA, New York Heart Association; T2D, type 2 diabetes mellitus; SGLT2I, SGLT2 inhibitor; oGLDs, other glucose-lowering drugs; NGAL, Neutrophil Gelatinase−associated Lipocalin; HbA1c, glycated hemoglobin; LVEF, left ventricle ejection fraction; LVEDP, Left ventricular end-diastolic pressure; SCr, serum creatinine; OAD, oral anti-diabetic agent; BNP, brain natriuretic peptide; ACS, acute coronary syndrome; NSAID, non-steroidal anti-inflammatory drugs.

Of the seven studies included in this systematic review, three were retrospective cohort studies (RCS) (24, 26, 27), two were prospective cohort studies (PCS) (22, 23), one was a case-control design (25), and one was an RCT (28). Three studies were conducted in Europe, two in Asia, and two in America. Of the seven studies included, only one reported the type, dose, and length of administration of the SGLT2I (28). One study reported the class but not the dose or length of the SGLT2I administered (24). Five studies did not report the type, dose, or duration of administration of the SGLT2I (22, 23, 25–27). At first, we excluded the study conducted by Bernardini F et al. (27) because this study compared a group of patients on “new-antidiabetic drugs” (including GLP-1 analogs, DPP-4 inhibitors, and SGLT2Is) vs. “standard therapy” (metformin, sulfonylureas, thiazolidinediones, and insulin) where the authors did not specify the number of patients on SGLT2Is neither specify the outcome of these group of patients.

This systematic review included 3,022 patients with type 2 diabetes mellitus undergoing CAG or PCI, 670 who received treatment with any SLGT2Is, and 306 CIN events. However, our meta-analysis included 2,572 patients, 512 of whom were treated with SGLTI2s and 289 acute renal failure events (**Table 1**). Most patients were in their sixties, with a minimum age of 45 and a maximum age of 80. The follow-up period varied from 1 to 24 months; however, four studies did not report the length of follow-up. Similarly, most studies did not report outcomes according to sex.

**Figure 2:**
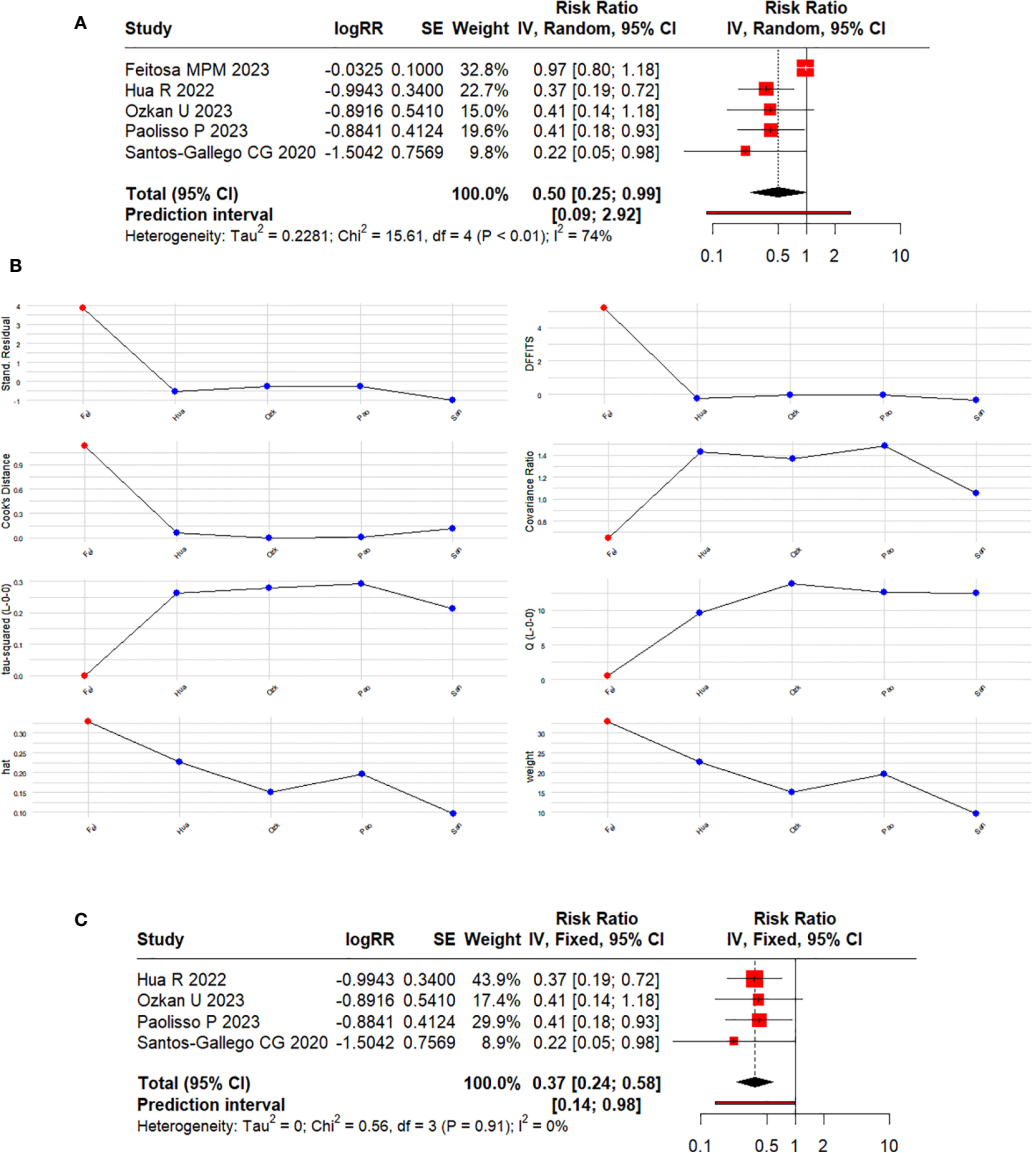
**(A)** Forest plot of the effect of SGLT2Is on the risk of developing CIN in patients with diabetes undergoing CAG or PCI considering non-duplicated (RCTs and observational) studies. **(B)** Plot of the influence analysis considering non-duplicated (RCTs and observational) studies included in the initial meta-analysis. **(C)** Forest plot of the effect of SGLT2Is on the risk of developing CIN in patients with diabetes undergoing CAG or PCI considering only observational studies.

Initially, we conducted a meta-analysis, including five studies (fourth observational and one RCT), obtaining an overall estimate (RR 0.50; 95% CI 0.25–0.99) for the risk of CIN among users of SGLT2Is compared to non-users. Nonetheless, the heterogeneity (I^2^ = 74%, *p* < 0.01) was unacceptably high (**Figure 2A**). We did not conduct subgroup, heterogeneity, or meta-regression analyses due to the scarce number of studies included, which may influence the robustness and ability to obtain significant and reliable results with these methods (29, 41–43). In the sensitivity and influence analysis (**Figure 2B**), we found that the study by Feitosa MPM et al. (28) had extreme values (outliers) that significantly impacted the overall estimate. Consequently, we excluded this study from our final meta-analysis (**Figure 2C**).

According to our results, SGLT2Is could reduce the risk of developing CIN by 63% (RR 0.37; 95% CI 0.24–0.58) in patients with diabetes undergoing CAG or PCI (**Figure 2C**), compared to those not using SGLT2Is. Statistical heterogeneity was not significant (I^2^ = 0%, *p* = 0.91).

Of the seven studies included in the systematic review, all six observational studies had a low risk of bias, according to the NOS tool. However, the only RCT included showed some concerns regarding the domain of “performance bias” due to the absence of blinding of participants and personnel and blinding of outcome assessment. Furthermore, the time of the patient’s exposure to SGLT2Is was short (30 days) (28).

*GRADE assessment.* We upgraded the level of CoE as all the studies included in the meta-analysis showed a low risk of bias (**Table 2**). Indirectness (the included studies compared similar interventions, similar populations, and similar outcomes), imprecision (this meta-analysis included 2,572 patients with diabetes undergoing CAG or PCI, 512 SGLT2Is users, and 289 events of CIN), publication bias, and inconsistency (I^2^ = 0) did not impact significantly the CoE. Nonetheless, the number of studies included was small. Then, we assessed the CoE according to GRADE criteria as moderate.

**Table 2:** Risk of bias of the included studies.

Author, study, country	Study design	Tool	Conclusion
Paolisso P et al. (22) 2022	PCS	NOS	Low risk
Paolisso P et al. (23) 2023,	PCS	NOS	Low risk
Hua R et al. (24) 2022	RCS	NOS	Low risk
Özkan U et (25). 2023	CCS	NOS	Low risk
Bernardini F et al. (27) 2022	RCS	NOS	Low risk
Santos-Gallego CG et al. (26) 2020	RCS	NOS	Low risk
Feitosa MPM et al. (28) 2023	RCT	RoB 2	Some concerns

PCS, prospective cohort study; RCS, retrospective cohort study; CCS, case-control study; RCT, randomized controlled trial; NOS, Newcastle–Ottawa scale (NOS); RoB 2, version 2 of the Cochrane tool for assessing risk of bias in randomized trials.

**Figure 3:**
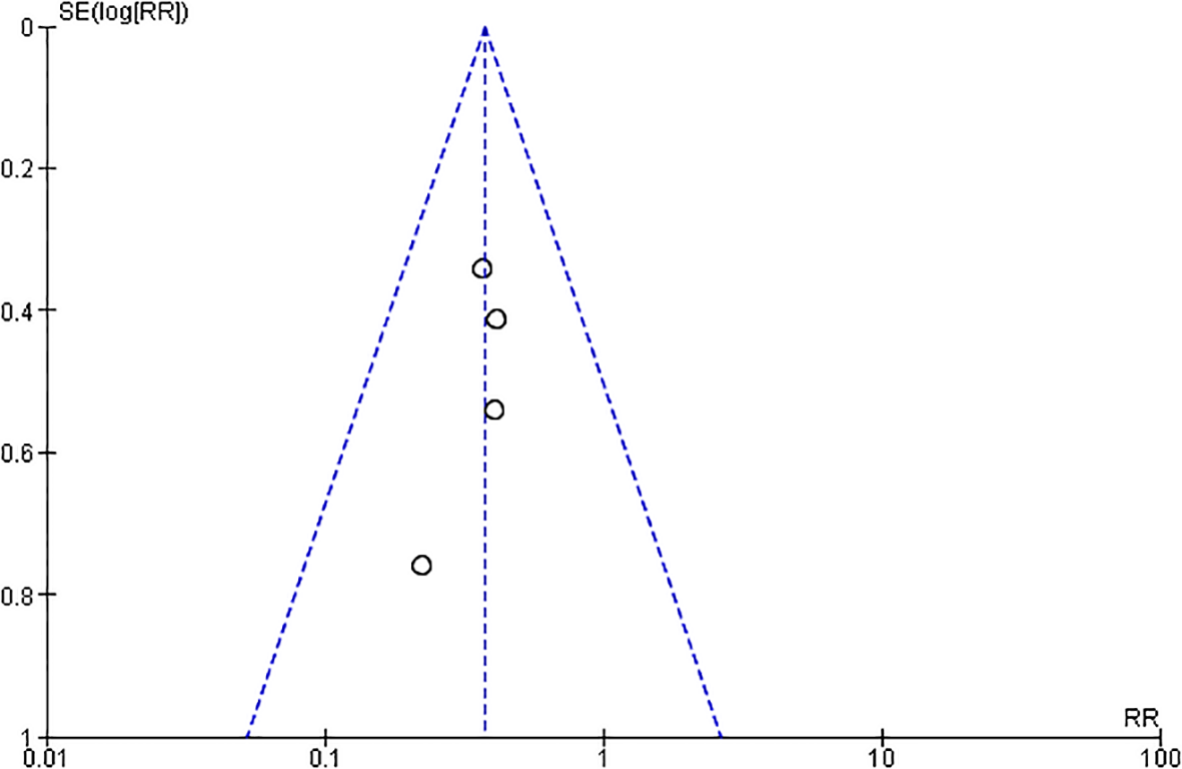
Funnel plot of the included studies in the meta-analysis on the effect of developing CIN in patients with diabetes undergoing CAG or PCI considering only observational studies.

The funnel plot of the included studies in our final meta-analysis did not suggest a publication bias (**Figure 3**).

## 4 Discussion

To our knowledge, this is the first systematic review and meta-analysis to investigate the effect of SGLT2Is on the risk of CIN in patients with diabetes undergoing CAG or PCI. Our findings suggest that SGLT2Is could reduce the risk of developing CIN by up to 63% (RR 0.37; 95% CI 0.24–0.58) in this population.

Since no previous systematic reviews have evaluated our PECO/PICO question, comparing our findings with similar studies is impossible. However, previous evidence derived from observational studies and RCTs is consistent with our results, which have shown that SGLT2Is reduce the risk of CIN in patients with diabetes undergoing invasive cardiac procedures with contrast administration (22–26).

The observational study conducted by Bernardini F et al. reported that patients with diabetes undergoing PCI and in treatment with new OADs (GLP-1 analogs, DPP-4 inhibitors, SGLT2Is) had a reduced incidence of CIN compared to patients with diabetes treated with traditional OADs (metformin, sulfonylureas, thiazolidinediones, insulin). Although this finding did not reach statistical significance (RR 0.7143; 95% CI 0.2324-2.1953), the authors concluded that their study underlined a possible protective role of new anti-diabetic drugs for preventing CIN (27).

Conversely, the only RCT assessing the effect of SGLT2Is on kidney function in patients with diabetes submitted to elective PCI was conducted in Brazil by Feitosa MPM et al. The SAFE−PCI trial was a prospective, open−label, randomized, single−center pilot study with a follow−up of 30 days. But, the objective of this study was quite different from the other studies included in our meta-analysis. They aimed to evaluate the safety of empagliflozin in these patients regarding kidney function. The researchers reported that the incidence of CIN, in the SGLT2Is group was 13.6% and 10.0% in the control group. However, this difference was not statistically significant (calculated RR 0.9680; 95% CI 0.7957-1.1776). They concluded that empagliflozin was safe regarding kidney function during elective PCI in patients with T2D compared with no SGLT2Is (28).

Even though the study by Feitosa MPM et al. was an RCT, we excluded it from our meta-analysis due to several reasons: 1) due to the heterogeneity caused when this study was included, 2) the small sample (22 patients in the intervention group and 20 patients in the control group), 3) concerns regarding the risk of bias due to the absence of blinding of participants, research staff, and outcome assessment, and 4) the short time of exposure to SGLT2Is i.e., “at least 15 days before PCI” (22). Indeed, several studies defined “exposure” to SGLT2Is as a cut-off value of six months because these drugs may render their cardiorenal beneficial effects for patients with T2D after this period (13, 24, 44).

Apart from the previously mentioned study, no other RCT has evaluated the nephroprotective effect of SGLT2Is in patients with diabetes after PCI or CAG. However, at least 8 RCTs are in different implementation stages aiming at answering this question (**Supplementary Materials**). Nevertheless, some RCTs have shown a renoprotective effect of SGLT2Is in patients with diabetes not undergoing contrast-enhanced coronary procedures. Two multi-center RCTs, the Empagliflozin, Cardiovascular Outcomes, and Mortality in Type 2 Diabetes (EMPA-REG OUTCOME) study and, subsequently, the CANagliflozin cardioVascular Assessment Study (CANVAS), demonstrated a decrease in the incidence of cardiovascular events and mortality with empagliflozin and canagliflozin, respectively (45, 46). Furthermore, these trials have shown that the use of Canagliflozin, Empagliflozin, or Dapagliflozin improved renal outcomes and is associated with slower progression of kidney disease and reduced the need for renal-replacement therapy in T2D (11, 14–16, 46–49).

Patients treated with SGLT2Is may have better kidney function than controls, reducing the baseline risk of CIN and inducing bias in the results of these studies. Of the five studies initially included in our meta-analysis, in the studies by Paoliso et al. (22, 23) and Hua R et al. (24), the patients in the group treated with SGLT2Is were younger. They also had better kidney function than the controls. But, there was no difference between the intervention groups in two other studies, Özkan U et al. (25) and Santos-Gallego CG et al. (26). Similarly, in the study by Feitosa MPM et al. (28), there were no differences in kidney function in both groups. In contrast, in the Bernardini F et al. (27) study, patients treated with SGKT2I had worse kidney function. As commented above, we excluded the last two studies from our final meta-analysis (27, 28).

Although the exact mechanisms by which SGLT2Is protect the kidneys are not fully understood, recent research has shown that these medications have numerous potential nephroprotective effects and traditional hypoglycemic effects. First, SGLT2Is cause osmotic diuresis, which leads to increased volume depletion. This volume depletion may help to protect the kidneys from contrast-induced injury. Second, SGLT2Is reduce renal medullary hypoxia, a significant risk factor for CIN. Third, SGLT2Is reduce glomerular hyperfiltration, decreasing excessive stress on the glomeruli and renal tubules, which could potentially lead to kidney damage over time. Fourth, these drugs have anti-inflammatory, antifibrosis, and antioxidant properties, which may also help to protect the kidneys from contrast-induced injury. Fifth, other proposed mechanisms are enhancing erythropoietin production, improving mitochondrial energy supply, inhibiting the sympathetic nervous system, protecting vascular endothelial cells, and reducing blood uric acid levels, among others (50, 51).

Our study has several strengths. First, we conducted a broad search strategy, including six important databases and registers of clinical trials. Second, we used a rigorous methodology to conduct our review and meta-analysis, including an exhaustive quality assessment of studies and a statistical analysis that accounted for heterogeneity. Third, the lack of statistical heterogeneity suggests that our findings are reliable and quite robust and that the results of individual studies are consistent.

Our study also has some limitations. First, a few completed studies have focused on explicitly answering our PECO/PICO question. Second, all the studies included in our meta-analysis were observational. Observational studies are more prone to bias than RCTs. Third, all the studies included in our meta-analysis were conducted in patients with type 2 diabetes. It is, therefore, possible that our findings may not be generalizable to patients with type 1 diabetes or patients without diabetes. Fourth, most studies included did not specify the type, dose, and duration of SGLT2Is administration. Consequently, performing subgroup analyses based on these factors was impossible.

## 5 Conclusions

Our findings indicate that SGLT2Is may significantly reduce the risk of CIN by up to 63% in patients with diabetes undergoing CAG or PCI. However, further RCTs are needed. Several RCTs are underway, aiming to confirm our findings and investigate other unresolved issues. These include determining whether SGLT2Is should be discontinued before CAG or PCI and identifying the optimal dose, type, and duration of SGLT2I therapy to prevent CIN, regardless of the presence or absence of diabetes or baseline kidney function.

## Data availability statement

The raw data supporting the conclusions of this article will be made available by the authors, without undue reservation.

## Author contributions

EM: Conceptualization, Data curation, Formal Analysis, Funding acquisition, Investigation, Methodology, Project administration, Resources, Software, Supervision, Validation, Visualization, Writing – original draft, Writing – review & editing. MA: Data curation, Investigation, Validation, Visualization, Writing – review & editing. GV: Data curation, Formal Analysis, Investigation, Methodology, Software, Validation, Visualization, Writing – review & editing.
