# Supplementary material for: SGLT-2 inhibitors and prevention of contrast-induced nephropathy in patients with diabetes undergoing coronary angiography and percutaneous coronary interventions: systematic review and meta-analysis
Source: Front Endocrinol (Lausanne). 2023 Dec 20;14:1307715. doi: 10.3389/fendo.2023.1307715 (PMC10765513; doi:10.3389/fendo.2023.1307715)
Supplement: Supplementary file 1 [file Table_1.docx]

**Table S1- Search strategy**

| **1. MEDLINE VIA PUBMED** | | **Results** | **Date** |
| --- | --- | --- | --- |
| #1 | "Sodium-Glucose Transporter 2 Inhibitors"[Mesh] | 5,364 | 22/September/2023 |
| #2 | "Contrast Media"[Mesh] | 95,620 |  |
| #3 | "Acute Kidney Injury"[Mesh] | 55,277 |  |
| #4 | #1 AND #2 AND #3 | 2 |  |
| **2. GOOGLE SCHOLAR** | | **Results** | **Date** |
| #1 | "Sodium-Glucose Transporter 2 Inhibitors" | 2,000 | 22/September/2023 |
| #2 | "Contrast induced nephropathy" | 30,100 |  |
| #3 | #1 AND #2 | 11 |  |
| **3. SCOPUS** | | **Results** | **Date** |
| #1 | "Sodium-Glucose Transporter 2 Inhibitors" | 4,109 | 22/September/2023 |
| #2 | "Contrast induced nephropathy" | 4,994 |  |
| #3 | #1 AND #2 | 3 |  |
| **4. COCHRANE LIBRARY** | | **Results** | **Date** |
| #1 | "Sodium-Glucose Transporter 2 Inhibitors" | 734 | 22/September/2023 |
| #2 | "Contrast induced nephropathy" | 997 |  |
| #3 | #1 AND #2 | 1 |  |
| **5. EMBASE** | | **Results** | **Date** |
| #1 | 'sodium glucose cotransporter 2 inhibitor'/exp | 22,620 | 30/March/2023 |
| #2 | 'contrast induced nephropathy'/exp | 16,757 |  |
| #3 | #1 AND #2 | 25 |  |
| **6. WEB OF SCIENCE** | | **Results** | **Date** |
| #1 | "Sodium-Glucose Transporter 2 Inhibitors" | 264 | 30/March/2023 |
| #2 | "Contrast induced nephropathy" | 3,864 |  |
| #3 | #1 AND #2 | 1 |  |

**Table S2.** Excluded studies and the reason for their exclusion

| **Study** | **Reason for exclusion** |
| --- | --- |
| Huang X, Guo X, Yan G, Zhang Y, Yao Y, Qiao Y, Wang D, Chen G, Zhang W, Tang C, Cao F. Dapagliflozin Attenuates Contrast-induced Acute Kidney Injury by Regulating the HIF-1α/HE4/NF-κB Pathway. J Cardiovasc Pharmacol. 2022 Jun 1;79(6):904-913. doi: 10.1097/FJC.0000000000001268. | Population and outcome different from our PECO / PICO question. |
| Nusca, Annunziata MD, PhD; Piccirillo, Francesco MD; Viscusi, Michele Mattia MD; Giannone, Sara MD; Mangiacapra, Fabio MD, PhD; Melfi, Rosetta MD; Ricottini, Elisabetta MD, PhD; Ussia, Gian Paolo MD; Grigioni, Francesco MD, PhD. Contrast-induced Acute Kidney Injury in Diabetic Patients and SGLT-2 Inhibitors: A Preventive Opportunity or Promoting Element? Journal of Cardiovascular Pharmacology 80(5):p 661-671, November 2022. \| DOI: 10.1097/FJC.0000000000001329 | Review. Only abstract is available. |
| Perlman A, Heyman SN, Stokar J, Darmon D, Muszkat M, Szalat A. Clinical Spectrum and Mechanism of Acute Kidney Injury in Patients with Diabetes Mellitus on SGLT-2 Inhibitors. Isr Med Assoc J. 2018 Aug;20(8):513-516. | Case series. |
| Sarafidis P, Papadopoulos CE, Kamperidis V, Giannakoulas G, Doumas M. Cardiovascular Protection With Sodium-Glucose Cotransporter-2 Inhibitors and Mineralocorticoid Receptor Antagonists in Chronic Kidney Disease: A Milestone Achieved. Hypertension. 2021 May 5;77(5):1442-1455. doi: 10.1161/HYPERTENSIONAHA.121.17005. | Review. |
| Lee B, Holstein-Rathlou NH, Sosnovtseva O, Sørensen CM. Renoprotective effects of GLP-1 receptor agonists and SGLT-2 inhibitors-is hemodynamics the key point? Am J Physiol Cell Physiol. 2023 Jul 1;325(1):C243-C256. doi: 10.1152/ajpcell.00147.2023. Epub 2023 Jun 5. PMID: 37273240. | Review. |
| Wu J, Cai H, Xia J, Zhang T, Yan J, Shao X, Wang AY, Shen J, Mou S. SGLT2i: Therapeutic options or contributing factors of contrast-induced acute kidney injury in the setting of non-diabetes? Pharmacol Res. 2023 Sep;195:106883. doi: 10.1016/j.phrs.2023.106883. Epub 2023 Aug 5. PMID: 37544050. | Letter to the Editor. |
| Chu C, Lu YP, Yin L, Hocher B. The SGLT2 Inhibitor Empagliflozin Might Be a New Approach for the Prevention of Acute Kidney Injury. Kidney Blood Press Res. 2019;44(2):149-157. doi: 10.1159/000498963. Epub 2019 Apr 2. PMID: 30939483. | Review. |
| Rampersad C, Kraut E, Whitlock RH, Komenda P, Woo V, Rigatto C, Tangri N. Acute Kidney Injury Events in Patients With Type 2 Diabetes Using SGLT2 Inhibitors Versus Other Glucose-Lowering Drugs: A Retrospective Cohort Study. Am J Kidney Dis. 2020 Oct;76(4):471-479.e1. doi: 10.1053/j.ajkd.2020.03.019. | The population is different from what was considered in our PECO question. |
| Patouliasa D, Papadopoulos D, Siskosa F, Doumasa M. Acute kidney injury with sodium-glucose co-transporter-2 inhibitors across the cardiovascular and renal outcome trials: Foe or friend? nefrologia 2023; 4 3(5):643–662 DOI: 10.1016/j.nefro.2021.05.012 | The population is different from what was considered in our PECO question. Letter to the Editor. |
| Lunati ME, Cimino V, Gandolfi A, Trevisan M, Montefusco L, Pastore I, Pace C, Betella N, Favacchio G, Bulgheroni M, Bucciarelli L, Massari G, Mascardi C, Girelli A, Morpurgo PS, Folli F, Luzi L, Mirani M, Pintaudi B, Bertuzzi F, Berra C, Fiorina P. SGLT2-inhibitors are effective and safe in the elderly: The SOLD study. Pharmacol Res. 2022 Sep;183:106396. doi: 10.1016/j.phrs.2022.106396. | The population is different from what was considered in our PECO question. |
| SGLT2 Inhibitors Prophylaxis Against Post-contrast Acute Kidney Injury in Diabetic Kidney Disease?  ClinicalTrials.gov ID NCT04853615 | Clinical trial. Not yet recruiting. |
| Dapagliflozin in the Prevention of Post-Coronary Angioplasty Acute Kidney Injury (DAPA-PCI-AKI)  ClinicalTrials.gov ID NCT05435235 | Clinical trial. Suspended. |
| proMoting Effective Renoprotection in Cardiac sURgery Patients by Inhibition of SGLT-2 (MERCURI-2)  ClinicalTrials.gov ID NCT05590143 | Clinical trial. Recruiting. |
| SGLT-2 Inhibitors in Prevention of Post-procedural Renal and Cardiovascular Complications aFter PCI Among Patients With Diabetes Mellitus and Coronary Artery Disease: a Prospective, Randomized, Pilot Study (SAFE-PCI) (SAFE-PCI)  ClinicalTrials.gov ID NCT05037695 | Clinical trial. Unknown status. |
| iSGLT2 in Prevention of Acute Kidney Injury in Patients With Diabetes Mellitus Undergoing CABG Extracorporeal On-Pump (POST-CABGDM)  ClinicalTrials.gov ID NCT04523064 | Clinical trial. Not yet recruiting. |
| Empagliflozin on Cardiac-renal Injury in Patients With STEAMI Patients After Primary PCI  ClinicalTrials.gov ID NCT03591991 | Clinical trial. Unknown status. |
| Dapagliflozin to Prevent the Incidence of Contrast Induced Nephropathy After Heart Catheterization and Percutaneous Coronary Intervention  ClinicalTrials.gov ID NCT04806633 | Clinical trial. Not yet recruiting. |
| The Effects of Empagliflozin on Renal Outcomes in Post Severe Acute Kidney Injury Survivors  ClinicalTrials.gov ID NCT05360615 | Clinical trial. Recruiting. |
